# Non-HIV talaromycosis: Radiological and clinical analysis

**DOI:** 10.1097/MD.0000000000019185

**Published:** 2020-03-06

**Authors:** Xinchun Li, Wenqing Hu, Qi Wan, Qiang Lei, Chongpeng Sun, Zhongjun Hou, Nitesh Shrestha

**Affiliations:** a Department of Radiology, First Affiliated Hospital of Guangzhou Medical University; b Department of Radiology, Second Affiliated Hospital of Guangzhou Medical University, Guangzhou, China.

**Keywords:** PET/CT, spiral CT, SUV, talaromycosis

## Abstract

To investigate the characteristics of spiral computed tomography (CT), positron emission tomography–computed tomography (PET/CT) and clinical manifestations of talaromycosis to improve the diagnostic level and deepen its recognition in radiology.

Radiological, clinical, and pathological manifestations of 15 patients of non-HIV talaromycosis confirmed by bronchofiberscope lung biopsy and/or abscess puncture fluid culture and/or blood culture and/or sputum culture were analyzed retrospectively. All patients underwent chest CT, among them, six had a brain MRI, and six had a PET/CT scan before treatment.

On plain CT scan, there were multiple patches and massive consolidation in 6 patients, multiple patchy consolidations and patchy ground-glass opacities in 3 patients, solitary or multiple nodules and masses in 3 patients, multiple cavities and small nodules in 3 patients. Multiple lymphadenectasis appeared in bilateral hila, mediastinum, and neck in 10 patients. In contrast CT scan, the parenchyma of the lesions had a slight enhancement in 10 patients, moderate enhancement in 3 patients, obvious enhancement in 2 patients. Seven cases had bone destruction and hyperplasia, cranial involvement in 1 patient and liver involvement in 3 patients, respectively. On PET/CT, five patients showed elevated standard uptake value (SUV).

The radiological manifestations of non-HIV talaromycosis show multiple consolidations, ground-glass opacities, multiple nodules or masses in bilateral lungs, deep-seated enlarged lymph nodes and bone destruction in multiple systems. The final diagnosis should be based on the culture of talaromycosis.

## 1 Introduction

Talaromycosis (formerly called Penicilliosis) is a rare deep soft tissue fungal infection caused by *Talaromyces marneffei* (formerly called *Penicillium marneffei*) fungi in the skin, lymph nodes, and visceral organs. It is a temperature dependent biphasic fungus, which was reported to infect patients firstly by Di Sal vo et al^[1]^ in 1973. Talaromycosis commonly occurs in patients with human immunodeficiency virus (HIV) and rare in patients with normal immunity. In recent years, the incidence of talaromycosis has gradually increased in non-HIV patients.^[2,3]^ Liu et al^[4]^ stated the radiological findings of bone involvement in non-HIV talaromycosis patients, mainly osteolytic bone destruction in flat bones. Zeng et al^[5]^ explored the clinical, pathological, and immunological features of 7 infants with non-HIV talaromycosis. Zhang et al^[6]^ first reported the radiological features of recurrent talaromycosis in the lungs showing multiple exudative shadows with thick-walled cavities, bilateral pleural, and pericardial effusion accompanied by multiple bone destruction in non-HIV patients in 2016. Deesomchok et al^[7]^ explored the radiological manifestations of the lungs in 12 patients with talaromycosis, including nine patients of HIV with talaromycosis, which has mainly diffuse pulmonary reticular and ground-glass changes, etc. However, there are rare reports on radiological features of the lungs in non-HIV talaromycosis.

In this study, radiological findings, clinical manifestations, pathological diagnosis, and prognosis to explore its radiological features of 15 patients of non-HIV talaromycosis confirmed by bronchofiberscope lung biopsy and/or abscess puncture fluid culture and/or blood culture and/or sputum culture were analyzed retrospectively.

## 2 Materials and methods

### 2.1 General clinical data

A retrospective study was performed in 15 patients of non-HIV talaromycosis confirmed by bronchofiberscope lung biopsy and/or abscess puncture fluid culture and/or blood culture and/or sputum culture from January 2010 to October 2017 in our hospital. There were 12 males and 3 females, the age ranging from 1.8 to 66.0 years old (mean 41.5 years). The follow-up period was from 2 months to 7 years. The serological tests of HIV antibody were negative at least one time in our hospital. Among them, 10 patients had negative-HIV antibodies for multiple times of serum tests. All the patients underwent radiological examinations with approval of the institutional review board of our hospital, and all the patients signed the informed consent form. All the patients have provided informed consent for publication of the study.

### 2.2 Equipment and examination methods

Fifteen patients underwent plain CT and enhanced CT examinations of the chest within a week after hospitalization. Also, 13 patients underwent abdominal CT examinations. The Toshiba Aquilion 16-slice spiral CT made in Japan, and Siemens Definition 128-slice spiral CT made in Germany were used to perform scanning of the chest and upper abdominal CT examinations in exposure factors of 120 kVp and 70 to 200 mAs. The scanning slice thickness was 0.5 mm, and the reconstruction slice thickness was 2.0 and 5.0 mm. During enhanced scanning examination, a bolus injection was mixed with non-ionic iodine contrast agent (300 mg·I/mL) in an amount of 1.5 mL/kg in a flow rate of 3 to 4 mL/s through the cubital vein. The CT data were evaluated by two senior attending radiologists.

On PET/CT of the whole body, GE Healthcare Discovery ST Hpower 60 made in the United States was performed in 6 patients, by using 18F-FDG in an amount of 5 MBq/kg as a tracer agent. After intravenous injection of 18F-FDG in 60 min, the images were acquired in 3D with scanning parameters of 140 kVp, 120 mAs, 3.75 mm in thickness and 0.875 in pitch. The standard uptake value (SUV) was measured at the maximal concentration slice of radioactivity.

The MRI examination of the heads was performed in 6 patients by using Philips Intera Nova Dual 1.5T superconducting dual gradient MR equipment made in the Netherlands for both plain and contrast scans. The scanning parameters were as follows: T1WI/SE (TR/TE = 500/15 ms), T2WI (TR/TE = 3732/100 ms), fluid low attenuation inversion recovery (FLAIR) (TR/TE = 8000/140), scanning thickness/interslice spacing = 5 mm/0.5–1 mm, field of view (FOV) in 23 to 25 cm and matrix in 512 × 512. On contrast scan of T1WI, scanning orientations were on axial, sagittal, and coronal planes and using Omniscan as a contrast agent in an amount of 0.2 mL/kg through the cubital vein.

## 3 Results

### 3.1 Clinical manifestations and routine laboratory tests

In all 15 patients of talaromycosis, there were cough and expectoration in 12 patients, fever in 10 patients (38–40.3°C, mean 38.9°C), hemoptysis in 1 patient, pain in the neck, chest, and back in 4 patients, subcutaneous mass in the neck, chest, and back or umbilical fossa-like rash in 7 patients (Table 1) and weight loss in 7 patients. Twelve patients had no chronic underlying diseases except for three patients with pulmonary tuberculosis, hypertension, and silicosis. There were 11 patients with elevated white blood cells count ([4.18–38.25] × 10^9^; mean [16.48 ± 8.66] × 10^9^), and 11 patients with increased neutrophil counts ([1.0–36] × 10^9^; mean [12.84 ± 8.43] × 10^9^). All the patients had decreased red blood cell counts ([2.49–4.43] × 10^12^; mean [3.55 ± 0.59] × 10^12^) and decreased hemoglobin test (70–114 g/L; mean 87.33 ± 11.05 g/L). The 2D dimer increased in 12 patients (12/12), and fungal 1–3-B-D dextran quantitative G test was positive in 8 patients (8/15).

**Table 1:**
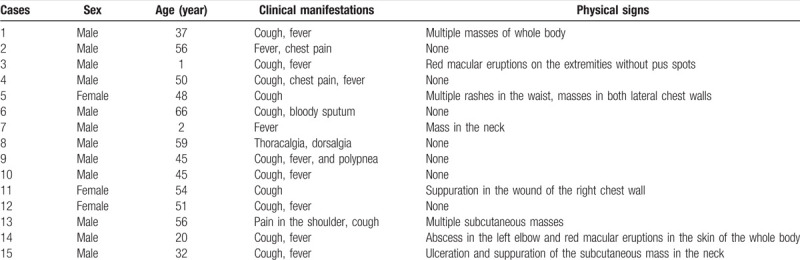
Clinical manifestations and signs of non-HIV PSM in 15 patients.

Fifteen patients were followed-up by CT several times during treatment. Before confirmed diagnosis, the pulmonary lesions became increased gradually. After the final diagnosis of talaromycosis, amphotericin B liposome was given intravenously with other anti-fungal medicine. The 15 patients were recovered and discharged with anti fungal drugs.

### 3.2 Radiological findings

**Figure 1:**
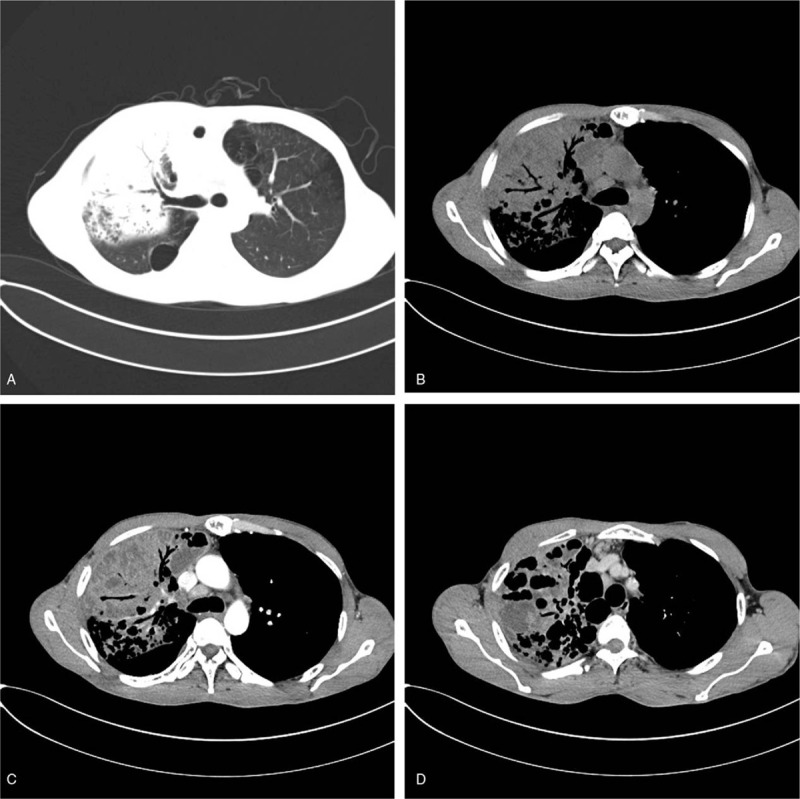
Male, 50 years old, recurrent cough and expectoration for more than 2 months, chest pain for 1 month and fever for 2 weeks. Lung window shows multiple large consolidations in the right lung with air bronchogram (A). On the mediastinal window, there are multiple cystic and patch-like fluid density areas and cavities and air spaces. Some of them display air–fluid levels. Multiple enlarged lymph nodes are shown with obvious inhomogeneous enhancement (B–D).

**Figure 2:**
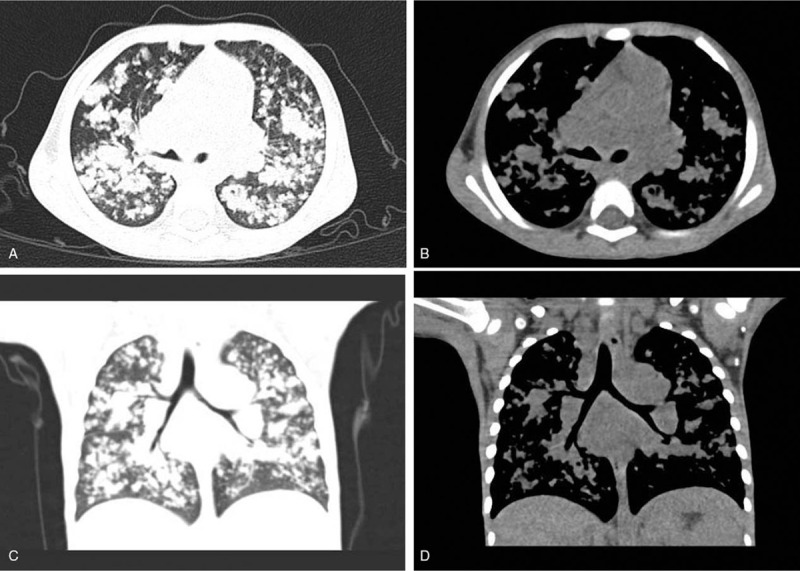
Male, 2 years old, recurrent fever for 1 month with aggravation and a cervical mass in 10 days. CT shows that both lungs have multiple nodular and patchy densities, which are mainly distributed along the bronchi in varied size. There is lymphadenectasis in both hila, mediastinum, bilateral supraclavicular regions and bilateral axilla. Among them, some enlarged lymph nodes are fused to form masses (A–D).

**Figure 3:**
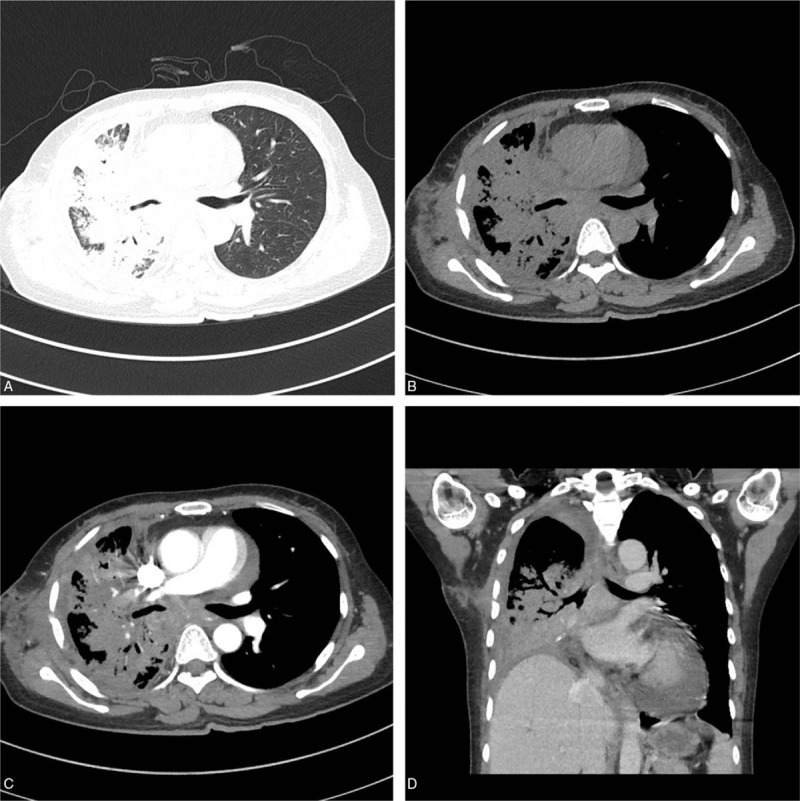
Female, 54 years old, recurrent cough and expectoration for 2 months (removed the right middle and lower lung lobes due to suspected central pulmonary cancer). CT shows post-operation changes of the right middle and lower lung lobes. Multiple consolidation and atelectasis happen in the right upper lung with stenosis of the right main bronchus. There are multiple enlarged lymph nodes in the right hilum and mediastinum with a little fluid in the right pleural cavity and thickening of the right pleura (A–D).

**Figure 4:**
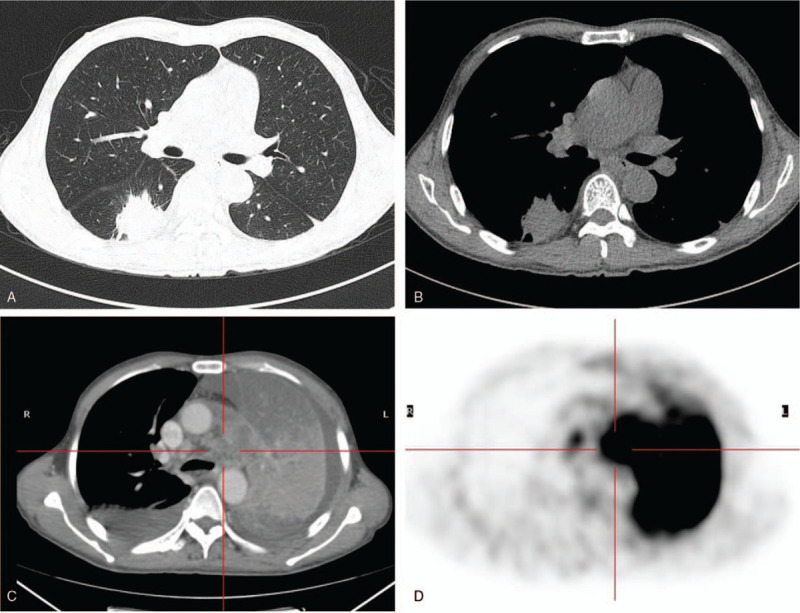
Male, 59 years old, repeated chest pain in a month and a massive opacity in the right lung for 3 weeks. CT shows a massive opacity with lobulation and speculation in the margin of the lesion, which has a uniform density and mild-moderate enhancement in the superior segment of the right lower lung (A and B). Four months later, the left lung shows large atelectasis with bilateral pleural effusion (C). PET/CT shows atelectasis in the left lung with a high concentration of radioactivity in the maximum SUV value of 19.9 (D).

In all 15 patients, 13 patients had multiple lesions in both lungs and solitary pulmonary lesions in 2 patients on CT examinations for the first time. There were multiple patches and massive exudative consolidation, mainly in 6 patients (6/15, 40%) (Fig. 1), multiple patchy exudative consolidations and patchy ground-glass opacities in 3 patients (3/15, 20%), solitary or multiple nodules and masses in 3 patients (3/15, 20%) (Fig. 2). The multiple cavities with an uneven thickness of the walls accompanied by small nodules were found in 3 patients (3/15, 20%). Two of them had huge cavities without obvious parietal nodule enhancement. Thirteen patients had a mild and moderate enhancement in the parenchyma of the lesion, while two patients had obvious enhancement. Larger lesions showed obvious necrosis without enhancement. There were air bronchogram in 5 patients, bronchial stenosis in 2 patients (Fig. 3), pericardial effusion in 4 patients, a little pleural effusion or pleural thickening in 6 patients. There were multiple lymphadenopathies in bilateral hila, mediastinum, and neck in 10 patients. The enlarged lymph nodes were partially fused and accompanied by multiple necroses. SUV elevated in 5 patients in a range of 2.5 to 13.9 (Fig. 4).

**Figure 5:**
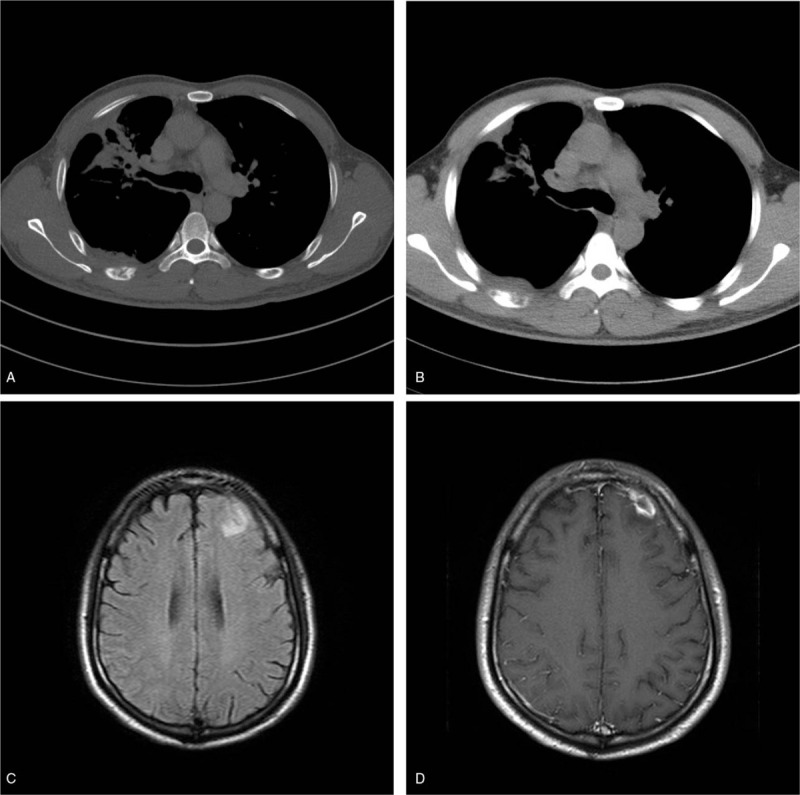
Male, 37 years old, recurrent cough and fever for 1 month. The new lesions appear in the right upper lung in massive opacities and cord-like shadows with bone destruction (A) and soft tissue mass (B) in the right sixth rib. MRI shows a patchy high signal in the left frontal lobe on T2WI/FLAIR (C). The lesion shows an annular enhancement (D).

Except for involvement of the lungs in talaromycosis, the lesions also involved bone in 7 patients, brain in 1 patient (Fig. 5), the liver in 3 patients, spleen in 2 patients. For bone involvement, it showed multiple osteolytic and worm-eaten like bony destruction with a slight hyperosteogeny mainly in flat bones and axial bones. In six patients with MRI examinations in the head, one patient had a solitary nodule in the left frontal lobe showed low signal on T1WI and high signal on T2WI and typical annular enhancement after contrast on T1WI whereas no abnormal findings were found in the remaining five patients. Involvement of the liver and spleen showed diffuse enlargement with scattered small nodular, circular, or nodular enhancement.

Initially, CT misdiagnosed 13 patients in all 15 patients (13/15), while whole body PET/CT misdiagnosed 4 patients out of 6 patients (4/6).

### 3.3 Diagnostic criteria of talaromycosis

**Figure 6:**
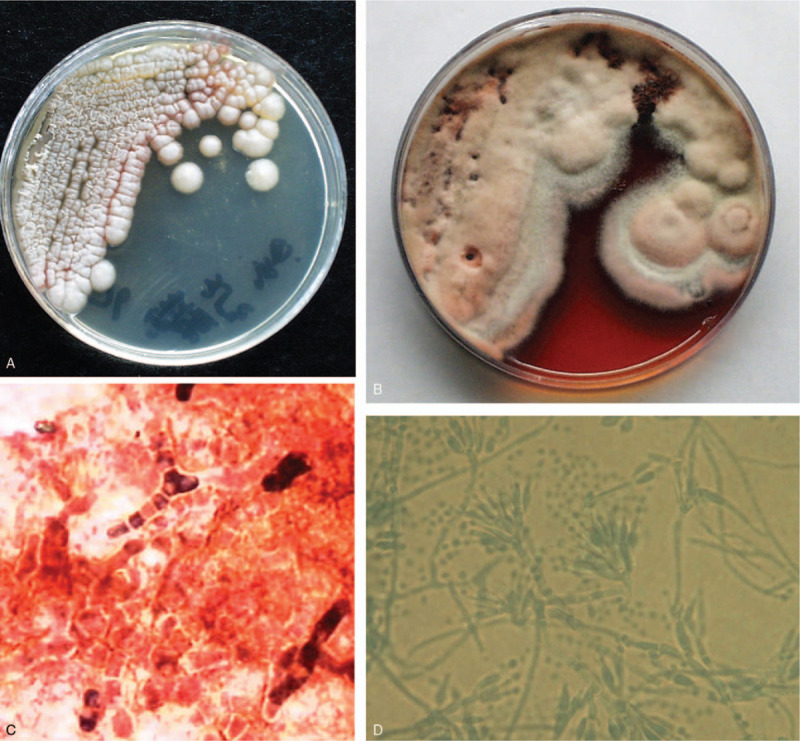
*Talaromyces marneffei* (37°C, yeast phase) has been incubated for 7 days to produce colony growth which looks like round, gray-white with a smooth surface and cut edge and without pigment in the Sabouraud's medium (A). *T marneffei* (25°C, Penicillium phase) has been incubated for 15 days, and the colonies turn into pale-yellow in the villous pattern, because a certain amount of soluble pigment makes the medium red (B). Taken a piece of cultured colonies at 37°C to make mount by direct compression, *T marneffei* is visible under the microscopy in high magnification (lactophenol cotton blue staining, ×400). *T marneffei* (yeast phrase) looks like a pattern of sausage (C). Taken a piece of cultured colonies at 25°C to make mount by direct compression, *T marneffei* is visible under the microscopy in high magnification (lactophenol cotton blue staining, ×400). The hypha of *T marneffei* (penicillium phase) looks like a broom in a diameter of 2 to 3 μm (D).

Fifteen patients of talaromycosis were finally diagnosed by culture of *T marneffei* by bronchofiberscopic lung biopsy in 2 patients, sputum culture in 2 patients, sputum and blood culture in 1 patient, abscess puncture fluid culture in 3 patients in superfical parts of the body, bronchofiberscopic sputum culture in 3 patients; sputum and abscess puncture fluid culture in superficial part of the body in 1 patient; blood culture in 2 patients and smear of sputum fungi finding of spores and hyphae (*T marneffei* as sausage pattern) in 1 patient. The diphasic fungal culture was performed in 15 patients for hyphal cells growth in the sputum, blood, or tissue. It took 3 to 7 days to product characteristic red-wine pigment in temperature of 25°C while the yeast spores were produced in temperature of 37°C in appearance of sausage under the microscope. Finally, *T marneffei* was diagnosed (Fig. 6).

### 3.4 Follow-up and prognosis

Fifteen patients were treated with various antibiotics before the final diagnosis of talaromycosis without any improvement; then, the patients were transferred to our hospital. Before confirmed diagnosis of talaromycosis, the lung lesions were gradually increased. After confirmed diagnosis of talaromycosis, all patients were treated with anti-fungal drugs, and the symptoms were relieved. On CT examinations of 13 patients, the lesions were absorbed and reduced. However, there were slight decrease in the huge cavity in 2 patients, but their clinical symptoms were significantly relieved. Among them, one patient had unilateral pulmonary nodules and developed a large consolidation in the contralateral side of the lung after 4 months (Fig. 4). Another patient presented with large and patchy consolidation with air bronchogram in the left lung for the first time. After 50 days of anti-fungal treatment, the lesions were reduced, and the patient was discharged. After 2 years, a patchy shadow occurred in his right upper lung because the patient stopped taking drugs by himself, and finally, talaromycosis was proved.

## 4 Discussion

Talaromycosis can be divided into the disseminated type and focal type. Disseminated type often involves lung, liver, spleen, lymph node as well as skin and other tissues and organs. In this study, 15 patients belonged to disseminated type, including 2 infants, 13 middle-aged and old people (12 males, 3 females). The clinical manifestations were complex, diverse, and lack of specificity. Kawila et al^[8]^ reported that clinical manifestations of talaromycosis with non-HIV were different from those talaromycosis with HIV. The patients with non-HIV talaromycosis had less fever and subcutaneous nodules or abscess in old people. However, ten patients (10/15, 66.67%) had a fever, seven patients (7/15, 46.67%) with subcutaneous nodules and abscess including one patient with silicosis and one patient with pulmonary tuberculosis in this study. It is suggested that subcutaneous nodules or abscesses in talaromycosis patients are not related to underlying lesions. There were 12 patients of talaromycosis without underlying diseases, and only three patients with underlying diseases, including one patient of pulmonary tuberculosis, one patient of hypertension, and one patient of silicosis. Qiu et al^[9]^ reported that clinical manifestations and prognosis of 43 non-HIV patients with talaromycosis in which 58.14% of them had no underlying lesions and 41.86% had underlying lesions, suggesting that the clinical manifestations of talaromycosis were not related to underlying lesions, but the imaging changes of lung lesions were not described. Hu et al^[2]^ reported that 668 patients with talaromycosis confirmed in China, among them 25 had underlying lesions (3.74%), 57 patients (8.53%) had no underlying lesions, and 586 patients (87.72%) accompanied with HIV. Out of the 15 non-HIV talaromycosis patients, 12 patients (80%) had no underlying lesions, which was significantly higher than that reported literature. Only three patients (20%) of talaromycosis had underlying lesions in our research, which were different from 85.7% with underlying lesions in non-HIV cases reported by Wong et al.^[10]^ Diabetes mellitus is the most common underlying lesion of talaromycosis. However, none of them had diabetes mellitus in this study.

Lou et al^[11]^ introduced that there was one patient of talaromycosis with systemic lupus erythematosus. In this case, multiple fine nodules scattered in the lung interstitials. Furusawa et al^[12]^ listed one patient of non-HIV talaromycosis with interstitial pneumonia. In this study, only one patient showed a focal pulmonary nodule initially, and the other 14 patients had evolved multiple patch-like shadows and a large consolidation in the lungs in the course of follow-up.

The radiological features of lung involvement in 15 patients with non-HIV talaromycosis are summarized as follows:

1. Lungs are initially involved and accompanied with multiple systemic lesions, including enlargement of multiple lymphatic nodes, bone destruction, involvement in the liver, spleen, and brain.
2. Bilateral lungs are affected, and multiple patches or huge consolidations or ground glass opacities are showed with air bronchogram. These were different from Supparatpinyo et al^[13]^ that the lung lesions of talaromycosis manifested interstitial nodules, reticular network in the lungs, but in consistent with Qiu et al^[14]^ report;
3. For multiple masses in the lungs, there are no obvious lobulated and spicula margins. The lesions have evident necrosis with varied contrast enhancement;
4. There are thick-walled or thin-walled cavities in the lungs. Qiu et al^[14]^ reported that five patients (35.7%) had cavities. In this study, three patients (20%) had thick-walled or thin-walled cavities in the lungs, which was slightly lower than the rate of the reported literature. Involvement of the main bronchi with atelectasis was relatively rare in the lungs with talaromycosis.^[15]^ In this study, the first CT scan in 1 patient showed a solitary nodule in the right lower lung. After antibiotic treatment, the lesion in the right lower lung disappeared, but the wall of the left main bronchus thickened and resulted in atelectasis of the left upper lung. The lesions were gradually disappeared after anti-fungal treatment compared with talaromycosis with HIV;
5. Multiple deep lymph nodes, mainly in the hila and mediastinum, can be fused and necrotic. Its solid parenchyma can have heterogenous slight and moderate enhancement. In this study, there was hilar and mediastinal lymph nodes enlargement in 10 patients (66.67%), and it was higher than that of Qiu et al^[14]^ paper. The enlargement of lymph nodes in talaromycosis with HIV is relatively rare;
6. Osteomyelitis is more common. Seven patients had bony destruction (7/15, 46.67%), and all of them were found in patients without chronic underlying diseases. It mainly involved the flat bones and axial bones, and typically mainly osteolytic destruction with a small amount of bone hyperplasia. Qiu et al^[14]^ reported that 14 patients had bone destruction (14/35, 40%) out of 35 patients in non-HIV talaromycosis, while there was no bone destruction in 65 patients of talaromycosis with HIV. Up to now, no report has been found in bone involvement in patients with HIV-positive talaromycosis. Therefore, talaromycosis associated with bone destruction occurs in non-HIV and normal immune host;
7. Involvement in the liver, spleen, and other organs, is mainly manifested as hepatosplenomegaly with focal or multifocal slightly low densities without enhancement.^[16]^ There was liver involvement in 3 patients, spleen involvement in 2 patients in this study. Whole body PET/CT scan found multiple low-density nodules with a radioactive concentration in maximum SUV of 7.2 to 13.9 in the liver of a patient. PET/CT manifestations of talaromycosis have not been seen in other literature yet;
8. There is a rare report about cerebral involvement of talaromycosis.^[17]^ MRI showed that the left frontal cerebral lobe had a cortical abscess with edema, annular enhancement, and adjacent meningeal thickening. This is consistent with the reports of talaromycosis with HIV in the central nervous system;
9. After anti-fungal treatment, lung lesions could be absorbed and reduced, but two patients with huge lung cavities had an only minor decrease of lesions. So, it suggests that huge cavity lesions have a slow absorption than a small cavity. The pathological changes of talaromycosis are often lack of specificity, somehow like tuberculous reaction or suppurative inflammation. The clinical manifestations and radiological findings of talaromycosis are varied, and it is easy to be misdiagnosed. In this study, CT misdiagnosed in 10 patients (10/15, 66.67%), and PET/CT misdiagnosed in 4 patients (4/6, 66.67%). These were mainly due to being lack of comprehensive analysis in radiological features of pulmonary lesions, enlarged lymph nodes, involvement in bones, liver and other organs, clinical data and laboratory indexes. Also, there were not enough recognition in non-HIV talaromycosis.

Rathakarn Kawila et al^[8]^ reported that the mortalities of talaromycosis with HIV and non-HIV talaromycosis were 20.7% and 29.4%, respectively. In this study, 15 patients with non-HIV talaromycosis were all alive, but the follow-up period is short and needs to be prolonged.

The radiological findings of pulmonary changes in talaromycosis are complicated. It should be differentiated from infectious diseases such as tuberculosis, pneumocystis carinii pneumonia, Aspergillus pneumonia, lymphoma, lung cancer, lymph node metastasis of lung cancer, etc.

## 5 Conclusion

The clinical manifestations of non-HIV talaromycosis have no specificity and develop rapidly. Early diagnosis and accurate treatment are the keys to reducing mortality. The possibility of talaromycosis infection should be considered whenever the patient has a fever of unknown origin, multiple patches, nodules or masses in the lungs, systemic lymphadenopathy accompanied by bone destruction, liver and spleen enlargement, etc. The final diagnosis should depend on the culture of *T marneffei*.

## Acknowledgments

It is our great honor to thank Ms. Renli Ceng, a technician for her wholehearted help in CT and MRI examinations for the patients with talaromycosis. We are eager to thank the Institution Review Board of the First Affiliated Hospital of Guangzhou Medical University for their instruction and support. Meanwhile, we would like to thank Qingsi Zeng, a senior radiologist, and professor of Radiology, to hold the post of guarantor of our paper for his high academic responsibility.

## Author contributions

**Conceptualization:** Xinchun Li, Qi Wan.

**Data curation:** Xinchun Li, Wenqing Hu, Zhongjun Hou.

**Investigation:** Wenqing Hu, Zhongjun Hou.

**Project administration:** Qi Wan, Chongpeng Sun.

**Resources:** Xinchun Li, Wenqing Hu.

**Software:** Qi Wan, Chongpeng Sun, Zhongjun Hou.

**Supervision:** Xinchun Li, Wenqing Hu, Zhongjun Hou.

**Validation:** Qiang Lei, Chongpeng Sun.

**Visualization:** Qi Wan, Qiang Lei.

**Writing – original draft:** Xinchun Li.

**Writing – review & editing:** Xinchun Li, Qiang Lei, Zhongjun Hou, Nitesh Shrestha.
